# Does big data require a methodological change in medical research?

**DOI:** 10.1186/s12874-019-0774-0

**Published:** 2019-06-17

**Authors:** Amke Caliebe, Friedhelm Leverkus, Gerd Antes, Michael Krawczak

**Affiliations:** 1 Institute of Medical Informatics and Statistics, Kiel University, University Hospital Schleswig-Holstein, Kiel, Germany; 2 Pfizer Deutschland GmbH, Berlin, Germany; 3 Institute for Medical Education (Senior Fellow), University Hospital, LMU Munich, Munich, Germany

**Keywords:** Digitalization, Big data, Scientific methodology, Correlation, Causality, Data quality, Hypothesis generation, Validation

## Abstract

**Background:**

Use of big data is becoming increasingly popular in medical research. Since big data-based projects differ notably from classical research studies, both in terms of scope and quality, a debate is apt as to whether big data require new approaches to scientific reasoning different from those established in statistics and philosophy of science.

**Main text:**

The progressing digitalization of our societies generates vast amounts of data that also become available for medical research. Here, the big promise of big data is to facilitate major improvements in the treatment, diagnosis and prevention of diseases. An ongoing examination of the idiosyncrasies of big data is therefore essential to ensure that the field stays congruent with the principles of evidence-based medicine. We discuss the inherent challenges and opportunities of big data in medicine from a methodological point of view, particularly highlighting the relative importance of causality and correlation in commercial and medical research settings. We make a strong case for upholding the distinction between exploratory data analysis facilitating hypothesis generation and confirmatory approaches involving hypothesis validation. An independent verification of research results will be ever more important in the context of big data, where data quality is often hampered by a lack of standardization and structuring.

**Conclusions:**

We argue that it would be both unnecessary and dangerous to discard long-established principles of data generation, analysis and interpretation in the age of big data. While many medical research areas may reasonably benefit from big data analyses, they should nevertheless be complemented by carefully designed (prospective) studies.

## Background

The progressing digitalization of our societies generates vast amounts of data that are also made available for secondary, mostly opportunistic, use by third parties. Already in the late 1990s, the term ‘big data’ was coined for this type of “high-volume, high-velocity and/or high-variety information assets that demand cost-effective, innovative forms of information processing that enable enhanced insight, decision making, and process automation” [1]. Regardless of the legitimacy of the latter assertions, it is indisputable that big data has pushed the boundaries in terms of data quality, analysis, manageability and interpretability as well.

Today, the analysis of big data is commonplace in the information and trading industry and starts to reach medical research as well. In medicine, big data is expected to facilitate major improvements of the treatment, diagnosis, and prevention of diseases. The Institute of Medicine (IOM) of the US National Academies of Science, Engineering, and Medicine referred to these developments as ‘Learning Healthcare Systems’, thereby succinctly summarizing the IOM’s vision of how “to transform the way evidence on clinical effectiveness is generated and used to improve health and health care” [2].

Since medical research classically proceeds through studies designed to answer specific questions, it is often hampered by high costs, long timescales, and insufficient sample sizes. By drawing upon resources that are readily available, big data appears to circumvent such bottlenecks and to reduce the success requirements to mere computing power. At the same time, it is widely presumed that, owing to the mere size of the technical and logistic challenges, the use of big data requires different scientific methodology or, as the IOM put it, “alternative research methodologies” [3]. We disagree with this assertion and will discuss below whether, from a philosophy of science point of view, a paradigm shift is apt in medical research, and whether long-established principles of data generation, analysis, and interpretation should be questioned - or even discarded - in the age of big data. Trying to put things into perspective, we will consider some fundamental methodological aspects of big data analysis in medical research and highlight how the latter contrasts with applications in industry. As we shall see, what benefits online companies does not automatically benefit science, and several precautions must be taken in our view to render big data analysis meaningful in the context of medical research.

## Main text

### Big data analysis may facilitate hypothesis generation

Scientific hypotheses arise through either deduction or induction. Deduction uses available knowledge, and sometimes empirical observations, to derive a new hypothesis by way of strictly deductive logic. A typical example in medicine is the expectation of a particular effect of a particular drug, based upon physiological and pharmacological theory. Induction, by contrast, draws solely upon past experience in that it combines current knowledge, actual observations, and subjective (as opposed to logical) reasoning to create new hypotheses. “We reason inductively when we infer that all Xs are Ys because all past observed Xs were also Ys.” [4] One consequence of this constraint is that universal statements (such as “All Xs are Ys.”) can never be verified by induction alone.

The above notwithstanding, inductive reasoning has played a central role in scientific research in the past, and its significance may well extend to big data. In fact, its conceptual relatedness to inductive reasoning explains why big data analysis is sometimes referred to as ‘hypothesis-neutral’ research [5]. On the other hand, since the use of big data in scientific research is often tantamount to agnostic searches of heterogeneous and unstructured data, the results therefore often lack the self-suggesting nature of the outcome of classical hypothesis generation.

### Every hypothesis requires evaluation

Regardless of its origin, every newly derived hypothesis must stand up to empirical scrutiny. Even though this requirement applies to scientific research in general, it appears particularly appropriate for hypotheses arising from big data analysis because “a research finding is less likely to be true […] when effect sizes are smaller; when there is a greater number and lesser preselection of tested relationships; where there is greater flexibility in designs, definitions, outcomes, and analytical modes” [6]. Undoubtedly, all these characteristics are more or less typical of big data. Moreover, since big data are ‘big’, they are easily misunderstood as automatically providing better results through smaller sampling error. That the gain in precision drawn from larger samples may well be nullified by the introduction of additional population variance and bias is then often not appreciated.

Popper highlighted that, for a hypothesis to count as scientific, it must be falsifiable [7]. His view has since become the formal cornerstone of statistical testing, where researchers aim to reject null hypotheses for the benefit of alternative hypotheses constituting their own scientific conviction. If a hypothesis cannot be falsified in repeated attempts, our belief increases that it might be true. Even then, however, the hypothesis is not proven because a proof would require use of external information. Note that this constraint only applies to universal statements (“All swans are white.”) which are, by far, the more interesting and important ones in medical research. Existence statements (“There are black swans.”) can of course be confirmed empirically.

**Fig. 1:**
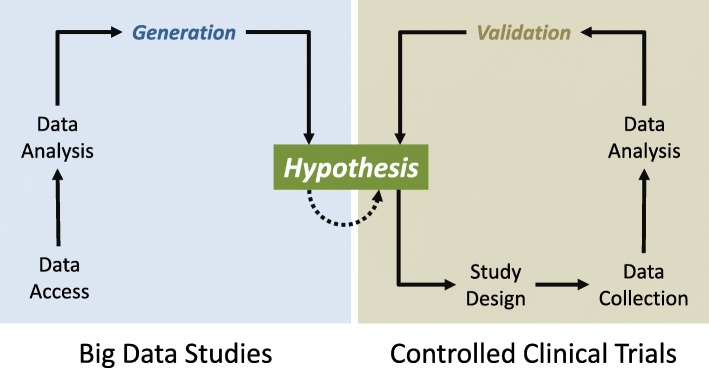
Comparison of big data studies and controlled clinical trials. Whereas big data studies (left inset) usually benefit from the opportunistic use of existing data resources, controlled clinical trials (CCT, right inset) follow a hypothesis-driven study design that determines the type, amount and provenance of the data to be collected. The subsequent data analyses may be methodologically similar or even identical, but the results of the two study types serve rather different purposes: The outcome of a big data study, at best, is a new hypothesis that would require verification in a CCT or controlled experiment to count as ‘scientific’ (dotted arrow). A CCT, by contrast, allows validation (i.e. falsification or verification) of the initial hypothesis, potentially stimulating further studies geared at the solidification, modification or diversification of this hypothesis

To test universal hypotheses, researchers must design studies that render falsification possible or, even better, likely (Fig. 1). The chance of falsification depends critically upon the specificity of the study design, the relevance and representativeness of the study subjects, and the quality of the data. It is therefore not surprising that experimental studies are the gold standard of scientific research, especially in disciplines such as physics and chemistry, which are founded upon experiments. In medicine, however, experimental studies are often logistically, legally, or ethically problematic if not impossible [8]. In such instances, well-designed observational studies are the next best option for the evaluation of hypotheses. Both approaches are, however, fundamentally different from the analysis of big data that originate outside the realm of control by the researcher and, hence, lack approved quality. In consequence, the bulk of scientific hypotheses derived through big data analyses require follow-up and validation by classical scientific studies before they can be deemed practically or theoretically relevant (Fig. 1).

### Causality versus correlation

“Correlation supersedes causation” [9] is one of the mantras of big data and, indeed, detecting and quantifying correlations may be a worthwhile exercise in various contexts. An online company, for example, would greatly benefit from knowing that customers who buy product X are also inclined to buy product Y. Whether this relationship is causal or not is irrelevant for the company to be economically successful. Similarly, in scientific research, being able to accurately predict an outcome of interest from correlated measurements can be useful, for example, because this may (i) point towards unknown causal mechanisms, (ii) help refining existing models of causality and (iii) provide a ‘reality check’ to the relevance of theories [10]. Moreover, as Mayer-Schönberger and Cukier [11] noted, “correlations may not tell us precisely why something is happening, but they alert us that it is happening.” Not surprisingly then, an area of medicine where correlation matters is the prediction of disease outbreaks. Even though the identification of causal effectors is an important goal of epidemiology, from a public health perspective, indirect but reliable outbreak markers may already be of great practical value.

The above notwithstanding, it is safe to say that the majority view in medical research ranks causality more important than correlation. In the words of Pearl [12], “most studies in the health sciences aim to answer causal rather than associative questions.” If a certain treatment is associated with a certain side effect, it is critical to know whether this relationship is causal because, otherwise, a change of therapy may be in vain. Similar reasoning applies to epidemiology where, despite the uncontested usefulness of prediction, the identification of causal risk factors is an important cornerstone of sensible public health policies. In most medical settings, correlation is therefore only the first step towards a mechanistic understanding of disease development and therapeutic effects.

Causality cannot be inferred from observational data alone but requires additional information or knowledge. From a purist point of view, only “discovering an underlying mechanism proves the truth of the causal hypothesis in question” [13]. It should be emphasized, however, that this extreme position is neither helpful nor necessary. In epidemiology, in particular, causality can often be inferred from observational data with reasonable certainty [13] and the Bradford-Hill criteria, first proposed > 50 years ago, still provide a good guide to transforming correlation into likely causality by way of combining different lines of evidence [14].

### The litmus test of big data: quality and quantity

Image analysis plays an important role in medical research where computer and magnetic resonance tomography scans, for example, easily qualify as big data, at least in terms of volume and velocity (two of the classical three Vs of big data, the other being variety). However, while processing of single images is an interesting research subject in its own right, particularly with a view to the use of machine learning and artificial intelligence techniques, the medically relevant content of an image often manifests at much lower levels of complexity. In the end, an image may comprise myriads of pixels, but is still one ‘measurement’. For instance, the task of classifying a mammogram as ‘tumour present’ or ‘tumour absent’ may require complex and computationally challenging algorithms, but the resulting diagnosis is essentially binary. Thus, inference making via medical image analysis may well draw upon big data, but most often it does not represent big data analysis sensu stricto.

In other research areas, particularly in clinical medicine, epidemiology and public health, big data are only of low to moderate quality because they are observational, uncontrolled, and only seemingly complete, of mixed origin and were not specifically collected for research purposes. In the context of biomedical research, Leonelli [15] identified three stages of the development of big data, namely de-contextualisation, re-contextualisation, and re-use. The author observed that “big data that is made available through databases for future analysis turns out to represent highly selected phenomena, materials and contributions. […] What is worse, this selection is not the result of scientific choices. […] Rather, it is the serendipitous result of social, political, economic and technical factors, which determines which data get to travel in ways that are non-transparent and hard to reconstruct […].” Note that this type of provenance is fundamentally different from what can be achieved in carefully designed clinical trials or experimental studies.

For big data, the famous idiom “garbage in, garbage out” may easily worsen to “big garbage in, big garbage out”. Data quality is an issue in medical research because of a frequent lack of standardization and structuring. Hence, any sensible use of big data in medical research critically depends upon the extent to which these data are ‘good data’ in the sense that everything possible has been done to sustain their quality. A fourth letter V was therefore proposed as an amendment of the three Vs of big data. The fourth V stands for veracity and for the challenge to ensure adequate care and diligence in big data analyses [16]. It is also worth emphasizing that small is sometimes beautiful in that ‘more data’ does not always imply ‘more information’, and that data reduction can be a sensible way to enhance data quality.

It seems paradoxical at first glance to regard insufficient sample size as a potential problem of big data. However, ‘big data’ often refers to large numbers of attributes but not necessarily to large numbers of independent observations of these attributes. In most commercial applications, such a limitation does not play a role because the data in question are gathered opportunistically from thousands of people anyway, usually as a by-product of internet-based services. In medical research, however, big data may comprise millions of attributes measured in a few patients, typically with the aim to provide them with ‘personalized medicine’. Schork [17] advocated this approach in a *Nature* commentary entitled “Personalized medicine: Time for one-person trials”. His views reflected the author’s particular professional interest in genomics, where millions of DNA variants are assessed in individual patients. However, it is a wide-spread albeit unfortunate misunderstanding that such ‘in-depth’ investigations of small samples ever lead to generalizable results. Instead, they should be deferred to the realm of case studies. The latter, however, are known to represent a low evidence level only and therefore have confined themselves mostly to hypothesis generation since the advent of evidence-based medicine in the 1990s.

## Conclusion: no change required

According to Kuhn [18], a paradigm shift involves a “crisis” and “pronounced failure” of prevailing theory, which is then “discarded” and “replaced” by new theory. As regards scientific reasoning, the introduction of big data into medical research clearly does not fall into this category. As was argued above, big data analysis may provide a means to potentially expand our scientific knowledge, but there is no reason why it should not comply with the established principles of scientific research. Unsustainable promises and unfulfillable expectations should be avoided in the context of big data and replaced by realistic views and evidence-based conclusions. Occasionally, the use of big data has been announced to take medical research “beyond evidence-based medicine” [19]. We do not subscribe to this view and believe that big data does not require an epistemic change in medical research.

That our considerations are not just mind games is exemplified by a much-noticed recent study by Khera et al. [20], published in the journal *Nature Genetics*, of so-called ‘polygenic risk scores’ (PRS). PRS are distilled from the summary statistics of large-scale genetic studies of common human disorders, in this case type 2 diabetes, coronary artery disease, inflammatory bowel disease, atrial fibrillation and breast cancer. Even though none of the scores was properly validated, neither prospectively nor by drawing upon existing long-term studies, and despite their obvious lack of predictive power [21], clinical use of the PRS for individual disease prediction has since been strongly advocated, both by the authors themselves [20] and by others [22].

In summary, we are adamant that obviating diligence and thoroughness in medical research with big data is a prospect that is apocalyptic, rather than paradisiac, and we would be wise to avoid it.

## Data Availability

Not applicable.

## Abbreviations

CCT: controlled clinical trial
IOM: Institute of Medicine of the US National Academies of Science, Engineering, and Medicine
PRS: polygenic risk scores
